# Electric Prostration

**Published:** 1890-07-12

**Authors:** 


### ELECTRIC PROSTRATION.

Some time ago, *vide* Retrospect, December 14th last, we referred to cases, reported chiefly from America, of injuries from electricity. It was then remarked that with the extended use of electricity a form of injury previously unknown, had now become sufficiently common to attract much attention, especially in America, where at present electricity is more used for various purposes than in this country. It now appears that such injuries are not the only consequences of the use of electricity, for Dr. A. Van Hoff Gosweiller, Physician to the Eastern Dispensary, has recently published an article (New York Med. Rec.) on "Electric Prostration." This, it is stated, results from exposure to the arc-light. The patient is apt to be awakened in the night by great pains around the orbits, accompanied with an out-pouring of tears, intense photophobia, and smarting pain. There is also a painful sensation in the throat, and the skin shows erythematous patches, afterwards peeling. The worst cases generally arise from adjusting the lamp (two thousand candle power) without goggles or smoked glass. The distance from the eyes averages about twenty inches, and one minute is sufficient to produce inflammation. Several cases of acute conjunctivitis have been reported consequent on exposure to the electric light, also cases of retinitis, but the effect on the throat and skin as detailed by Dr. Van Hoff Gosweiller has not been ordinarily noticed.

